# Lepra

**Published:** 1880-06-20

**Authors:** Jas. H. Low

**Affiliations:** New York City


### Southern Medical Record.

EDITORS:

T. S. POWELL, M.D. W. T. GOLDSMITH, M.D. R. C. WORD, M.D.

*R. C. WORD, M.D., Managing Editor.*

☞All Communications and Letters on Business connected with the RECORD must be addressed to the Managing Editor.

VOL. X. ATLANTA, GA., JUNE 20, 1880. No. 6.

### ORIGINAL AND SELECTED ARTICLES.

### LEPRA.

BY JAS. H. LOW, M. D., OF NEW YORK CITY.

Believing that the subjoined case might not be uninteresting to your numerous readers, I give you a synopsis of it.

We were called on the 12th of last December to see Mrs. N., residing on Fifty-ninth street in this city—a native of England, aged sixty-eight years—who has resided in the United States thirty years. When first seen, her limbs were swollen to twice their usual dimensions, and were of a dark, livid color, assuming that form of the disease denominated *Lepra Nigricans;* scales from one-quarter to one inch in diameter forming on her limbs to one pint or more in twenty-four hours, with general itching over the body and upper extremities; tongue heavily coated—edges and tip red; circulation from eighty to ninety per minute; little or no appetite, and very despondent, as she thought her case incurable—remarking that she had been treated for the last twelve months by doctors in Albany and New York city, who said they could cure her but had failed. She also complained of insomnia, caused principally by the stiffness and itching of the lower limbs, which were mostly involved at the beginning.

I endeavored to obtain from her the line of practice pursued by her former medical attendants, and as well as could be ascertained it was the usual course adopted in such cases, but as she claimed without any good results—hence her despondency. Under these circumstances we took charge of the case. We prescribed a pill composed of pilulæ hydgr., one grain; podophyllin, one-quarter of a grain; extract colocynth comp., one grain—one pill at bed-time. Also the bromide of potassium alternating with opiates to be taken *pro re nata* to quiet nervous excitement and procure sleep. We also prescribed liquor potassæ arsenitis in doses of eight drops three times daily; which treatment we continued ten days or two weeks; and while the pills and bromide acted like a charm, as evidenced in her satisfactory and refreshing sleep, and tongue cleaning off, and a return of appetite, yet the Fowler's solution produced that peculiar swelling—*œdema asenecalis*—which we discovered in her face and eyelids. She then informed us that some one of her former medical attendants had used that remedy before. We now commenced with the potassi iodidum in six-grain doses three times daily, in conjunction with the mercurial pills as we thought they were needed; and in consequence of the enormous swelling of the lower limbs, we applied equal-pressure roller bandages from the toes to half-way up the thighs. This treatment was pursued for two months; in the meantime, while the lower limbs seemed to improve a little, the upper extremities and whole body became involved. Language is inadequate to describe her appearance. I have not seen such a case in any of the hospitals I have ever visited. Hobbling to her bed, which she persisted in doing without help, she could be traced by the scales on the floor, handfuls being taken from her daily; in many instances the blood following the scraping off the scales. We had to discontinue warm baths. As the lower limbs first showed evidence of the disease and had continued up to the present, we concluded that a seclusion of Atmosphere, combined with equal pressure, was necessary to restore the blood-vessels to their normal condition; consequently, the starch bandage was applied twice per week to the lower limbs, and continuing the potassium iodide with the addition of the syr. sarsaparilla comp. three times a day—ten grains of iodide in two drachms of syrup. This treatment was continued for three months, observing a little improvement each successive week, the limbs gradually assuming their natural size and appearance with less of itching and scaly eruption. During the latter part of the treatment we gave quinine and iron as a tonic, and recommended driving in Central Park. In the latter part of our treatment erysipelas supervened, but disappeared by the application of usual remedies and the limb was left apparently freer from disease than before the attack of erysipelas. We persevered in the application of our starch bandage observing that Alcoholic stimulants internally or grease applied externally aggravate the symtoms. She now has a good appetite, tongue cleaned off, sleeps well, her skin smooth, her appearance natural and no symptom or trace of the disease left when we discharged her on the 25th of April last. She is now on a visit to friends in the upper part of this State.

NEW YORK CITY, May, 1880.

